# Dual Paper-Based Electrochemical Device for Multiplex Detection of Triple-Negative Breast Cancer miRNA Signatures

**DOI:** 10.1021/acs.analchem.5c07951

**Published:** 2026-05-22

**Authors:** Alessandra Glovi, Panagiota M. Kalligosfyri, Antonella Miglione, Sima Singh, Wanda Cimmino, Stefania Cocco, Umberto Malapelle, Antonio Giordano, Michelino De Laurentiis, Stefano Cinti

**Affiliations:** † Clinical and Translational Oncology, 9307 Scuola Superiore Meridionale (SSM), Naples 80138, Italy; ‡ Department of Pharmacy, University of Naples Federico II, Naples 80131, Italy; § Department of Public Health, 165485 University Federico II of Naples, Naples 80131, Italy; ∥ Department of Breast and Thoracic Oncology, 18730 Istituto Nazionale Tumori IRCCS “Fondazione G. Pascale”, Naples 80131, Italy; ⊥ Sbarro Institute for Cancer Research and Molecular Medicine, Center for Biotechnology, College of Science and Technology, 6558 Temple University, Philadelphia, Pennsylvania 19122, United States; # Department of Medical Biotechnologies, University of Siena, Siena 53100, Italy

## Abstract

In this technical note, we demonstrate how the merging of paper-based screen-printing, engineered probes, and multiplexing might offer a potent tool toward the analysis of cancer-related miRNA signatures. In fact, while single miRNA detection cannot be associated with a health condition, the detection of miRNA signatures (more than one sequence) offers a more comprehensive reflection of disease biology. The two working electrodes are engineered with rationally designed capture probes modified with two different redox mediators, namely methylene blue and ferrocene; this is necessary to discriminate against different miRNAs, i.e., miRNA-21 and miRNA-101, which are dysregulated in triple-negative breast cancer. The use of ferrocene and methylene blue as redox mediators enables independent, interference-free target detection within a single electrochemical system. The analytical characterization of the dual sensor for both analytes is consistent with the single systems, enabling their simultaneous detection in a wide concentration range (0.1–1000 nM). The platform achieves clinically relevant low-nanomolar limits of detection in both PBS and untreated human serum, while demonstrating excellent repeatability with a relative standard deviation of ∼4%, a rapid assay time of 30 min and straightforward operational procedures. Importantly, the proof-of-concept analysis performed in untreated spiked serum samples demonstrated reliable detection within a complex biological matrix. These findings highlight the potential of multiplex miRNA signature analysis in biofluids and warrant further evaluation in clinically relevant samples. Overall, this fast, low-cost paper-based platform enables multiplex analysis at the point of care and can be readily expanded to larger miRNA panels, providing a promising foundation for future diagnostic development.

## Graphical abstract

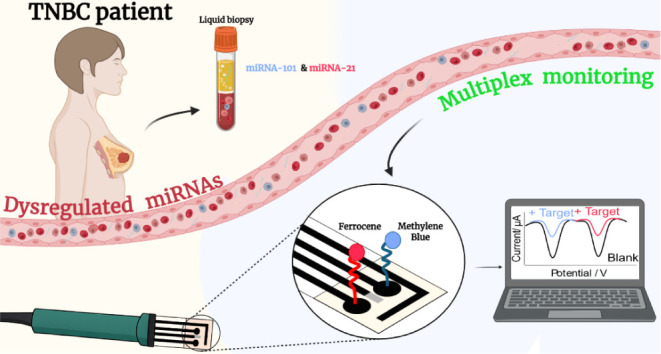

## Introduction

Although individual circulating miRNAs are promising cancer biomarkers due to their stability in body fluids and regulatory roles, their diagnostic performance is moderate when considered alone. Instead, miRNA signatures address these challenges by capturing multiple molecular pathways simultaneously, revealing the complex biological changes that occur during cancer development and treatment. Studies prove that combining two miRNAs for diagnostic purposes can achieve over 90% accuracy, surpassing the performance of single biomarkers.^1–3^ Multiplex analysis enables simultaneous measurement of multiple targets, highlighting intricate protein interactions underlying disease-specific dysregulation.^4,5^ Understanding cancer mechanisms, rather than simply detecting tumor cells, is particularly important in TNBC, which is highly heterogeneous. Multi-miRNA profiling provides valuable clinical insights for TNBC patients, given its genetic diversity and limited treatment options.^6–8^

To illustrate the value of multiplexed miRNA signatures in TNBC, two key circulating miRNAs, namely miRNA-21 and miRNA-101, form a complementary pair of markers. Their opposite expression patterns create a distinctive diagnostic signature that enhances detection accuracy beyond what either marker can achieve alone and provides a strong rationale for developing multiplexed diagnostic assays.^9^

The oncomiRNA miRNA-21 is one of the most consistently upregulated circulating miRNAs in TNBC and across several aggressive breast cancer subtypes.^10–13^ Its overexpression reflects the activation of multiple pro-survival and pro-inflammatory pathways,^14^ and it correlates strongly with tumor initiation, metastatic dissemination, and poor patient outcomes. Clinically, elevated circulating miRNA-21 has been repeatedly associated with worse prognosis and reduced therapeutic response, making it a robust indicator of TNBC progression. In contrast, the tumor suppressor miRNA-101 acts as a cancer inhibitor and is decreased in TNBC patients. Its levels are lower in TNBC tissues and cell lines, while restoring miRNA-101 reverses malignant traits.^15^ miRNA-101 limits prosurvival programs by targeting DNA damage–response kinases DNAPKcs and ATM, reducing double-strand break repair and sensitizing tumors to genotoxic stress. It also inhibits autophagy^16,17^ and suppresses antiapoptotic and pro-tumorigenic drivers, including MCL1 and CXCR7, reducing growth, invasion, metastasis, and enhancing paclitaxel-based therapy response.^18^

Detecting oncogenic miRNA-21 and tumor-suppressive miRNA-101 simultaneously enhances diagnostic accuracy, reduces technical errors, and reflects TNBC heterogeneity. This dual-marker approach enables circulating miRNAs to serve as practical biomarkers for diagnosis, treatment monitoring, and postsurgery follow-up. However, current centralized laboratory methods, such as quantitative reverse transcription polymerase chain reaction,^19^ microarrays,^20^ and next-generation sequencing,^21^ are unable to meet the demand for rapid, multiplexed miRNA detection in resource-limited and POC settings. Their reliance on complex, instrumentation-intensive workflows, including target amplification and precise temperature control, makes simultaneous detection of low-abundance circulating miRNAs difficult outside well-equipped laboratories. Fluorescent multiplex assays, for example, suffer from intrinsic limitations, such as overlapping emission spectra, signal instability, and the dependence on complex optical hardware, which further restrict their suitability for decentralized testing.^22,23^

Electrochemical detection methods offer a promising solution by providing built-in multiplexing functions through spatial, temporal and chemical discrimination methods, features that single-measurement techniques lack. In particular, redox-tag multiplexing methods allow the detection of multiple biomarkers simultaneously through potential-resolved voltammetry, because each target corresponds to a specific electrochemical mediator.^24,25^ Paper-based substrates for electrochemical sensors are well aligned with the simplicity required for POC applications.^26^ For example, miRNA detection assays have demonstrated high sensitivity; however, many rely on single-electrode probe immobilization, multistep surface modifications and reagent addition steps, or extended assay times under controlled elevated temperatures.^27,28^ In addition, only a limited number of studies in the past six years have focused on clinically relevant miRNA signature.^29^ Although paper-based electrochemical sensors offer clear advantages including simplicity, portability, and low cost, existing approaches still present important limitations. These constraints highlight the need for simpler, portable, and scalable strategies capable of accurate multitarget detection, while avoiding complex procedures and reliance on centralized laboratory infrastructure, thereby enabling more practical and effective POC implementation.

Building on these needs, we developed a dual-channel, screen-printed, paper-based electrochemical sensor for the simultaneous detection of miRNA-21 and miRNA-101, offering a practical platform for POC miRNA diagnostics. To demonstrate the value of multiplexed miRNA signatures in TNBC, we selected this biologically complementary pair of circulating miRNAs. miRNA-21 is frequently overexpressed in TNBC and associated with pro-survival signaling, invasion, and poor prognosis, whereas miRNA-101 functions as a tumor suppressor and is commonly downregulated.^15,30–32^ Together, this oncogenic–tumor suppressor combination provides a mechanistically informative dual-marker signature that reflects complementary molecular alterations in TNBC biology.

This work’s design addresses key technological and biological limitations associated with conventional single-marker assays. By combining dual-marker analysis, the platform reduces potential misclassification and improves diagnostic reliability through complementary biological information. Independent multiplex detection is achieved using ferrocene and methylene blue redox reporters with distinct electrochemical signatures, enabling interference-free signal differentiation and internal normalization.^33,34^ Integrated with AuNP-enhanced electrodes and paper-based sample processing, the platform offers a low-cost, portable, and disposable format for sensitive miRNA quantification. Furthermore, the system provides a scalable foundation for future expansion toward larger multiplex diagnostic panels.

## Experimental Section

### Materials

Potassium ferricyanide (K_3_[Fe­(CN)_6_]), hexaammineruthenium­(III) chloride (Ru­(NH_3_)_6_^3+^), chloroauric acid (HAuCl_4_), 6-mercapto-1-hexanol (MCH, C_6_H_14_OS), tris­(2-carboxyethyl)­phosphine (TCEP; C_9_H_15_O_6_P) and all the other common reagents were purchased from Sigma-Aldrich (St. Louis, MO, USA). Human serum (Hypo-Opticlear, Sigma-Aldrich) was used as received. The serum was not heat-inactivated, was stored at −20 °C upon arrival, and was thawed at room temperature (RT) immediately prior to use. The anti-miRNA-101 DNA probe tagged with methylene blue (MB) (5′-Thiol-C6-AGC ATC AGC ACT GTG ATA ACT G-MB-3′) (MB-tagged DNA probe) selective to miRNA-101, the anti-miRNA-21 DNA probe tagged with ferrocene (Fc) (5′-Thiol-C6-TCA ACA TCA GTC TGA TAA GCT A-Fc-3′) (Fc-tagged DNA probe) selective to miRNA-21, the target miRNA-101 sequence (5′-CAG UAA UCA CAG UGC UGA UCG U-3′) and the target miRNA-21 sequence (5′-UAG CUU AUC AGA CUG AUG UUG A-3′) were purchased from Metabion GmbH (Steinkirchen, Germany). Sequences tested as potential interferents, including miRNA-200 (5′-UAA CAC UGU CUG GUA ACG AUG U-3′) and miRNA-205 (5′-GAU UUC AGU GGA GUG AAG UUC-3′) were also sourced from Metabion GmbH (Steinkirchen, Germany), whereas miRNA-125 (5′-UCCCUGAGACUUGUGA-3′) was purchased from Biomers.net GmbH (Ulm, Germany). The dual-working-electrode configuration was manually screen-printed, following established procedures.^35^ The conductive silver ink was purchased from Loctite (Italy) and the carbon ink was purchased from Sun Chemical (USA).

### Apparatus

Adobe Illustrator Creative Cloud 2021 (Adobe Inc., San Jose, CA, USA) was used to design the wax pattern of the testing area of the paper-based screen-printed electrodes (SPEs). A solid ink printer, namely, ColorQube 8580 from Xerox (USA) was used to print the hydrophobic layer of wax onto paper-based substrates. Paper-based SPEs were fabricated in-house on office paper.^36^ All the electrochemical measurements were carried out using a portable potentiostat μStat ECL potentiostat (Metrohm DropSens Italia Srl, Origgio, Italy) and the results were visualized via a portable computer with the dedicated application DropView (8400).

### Fabrication of the Paper-Based SPEs

Paper-based SPEs were fabricated in-house using Fabriano office paper (80 g/m^2^) as the substrate, as previously described.^37^ Briefly, to define the hydrophobic barriers for fluid handling, a custom wax pattern was designed and printed using a Xerox ColorQube 8580 solid-ink printer. The printed sheet was then heated at 100 °C for 1 min to melt the wax and form the fluidic barriers. Subsequently, the dual-working-electrode configuration was obtained using dedicated masks by sequential manual screen-printing on the wax layer. First, silver ink was used to print the connections and the reference electrode. A subsequent graphite layer was printed to make the counter electrode and the two working electrodes (WEs) (2.5 mm in diameter each). After each printing step, the SPEs were cured at 60 °C for 30 min to remove the solvent and induce ink polymerization. The obtained electrochemical strips were approximately 2.5 cm in height and 1 cm in width.

### Functionalization of the Dual-WE Platform for miRNAs Detection

The functionalization procedure started with the modification of the two WEs by adding 2 μL of AuNPs per area via drop-casting. This approach enabled the simultaneous modification of multiple electrodes, allowing rapid and scalable production. The AuNPs, essential to enhancing the overall biosensor performance and for subsequent probe immobilization, were synthesized in our laboratory through an established protocol.^38^ The anti-miRNA-101 and anti-miRNA-21 DNA sequences were used as capture probes, with each probe immobilized onto its respective AuNP-modified WE. Each probe was functionalized with a redox mediator at the 3′ end: MB for anti-miRNA-101 and Fc for anti-miRNA-21. These redox labels were introduced to enable efficient electron transfer at the electrode surface. In addition, both probes were modified with a thiol at the 5′ end, enabling covalent binding to the AuNPs. Prior to immobilization, the anti-miRNA-101 and anti-miRNA-21 DNA probes (1 μM each) underwent thiol reduction in the presence of 10 mM TCEP for 1 h at RT. This step is essential for reducing disulfide bonds, activating the probe for covalent attachment to the AuNPs. Each reduced probe was then diluted to the desired concentration, i.e., 50 nM for the MB-labeled probe and 500 nM for the Fc-labeled probe. The MB-labeled probe concentration was selected based on previous optimization.^37^ For the Fc-labeled probe, 500 nM was chosen as the minimum concentration that provided a measurable and stable electrochemical signal, since lower concentrations did not yield reliable responses under the tested conditions. A volume of 10 μL of each solution was then applied onto the corresponding AuNP-modified WE for 1 h at RT in a humid chamber. The WEs were gently washed with distilled water and incubated in a humid chamber with 10 μL of 2 mM 6-mercapto-1-hexanol each for 1.5 h to passivate the empty spaces. Finally, the WEs were thoroughly washed again with double-distilled water to remove unbound thiols.

### Measurement of miRNA-101 and miRNA-21 Targets

All electrochemical measurements were performed at RT using a μStat ECL portable potentiostat with data acquisition and visualization carried out through the DropView 8400 software. Each paper-based SPE was measured individually. The signal was recorded using square-wave voltammetry (SWV) with the following parameters: equilibrium time = 5 s, E_start_ = 0.7 V, E_end_ = −0.7 V, E_step_ = 0.001 V, amplitude = 0.01 V, and frequency = 50 Hz. The selected potential window was chosen to fully encompass the oxidation peaks of ferrocene (+0.4 V) and methylene blue (−0.3 V), enabling simultaneous and interference-free detection of both redox mediators. The SPEs were initially assessed in the presence of 70 μL of a blank solution (PBS pH 7.4 or undiluted serum). The measurements were carried out 30 min after sample addition, as previously optimized,^38^ since this interval was determined to be the optimal time required to obtain a stable signal change in the presence of the target miRNA. For miRNAs detection, the droplets were spiked with the required concentration of the target miRNAs, and each measurement was taken after a 30 min hybridization period between the miRNAs and the anti-miRNA corresponding probe. The platform operates through a signal-off mechanism. In the absence of the target, the redox labels attached to the probes, methylene blue on one probe and ferrocene on the other, remain flexible and close to the electrode surface, allowing efficient electron transfer and generating a strong electrochemical signal. Upon hybridization with the complementary miRNA, duplex formation restricts the mobility of the redox label and increases its distance from the electrode, which diminishes electron-transfer efficiency and results in a decreased current (Figure 1).

**Figure 1:**
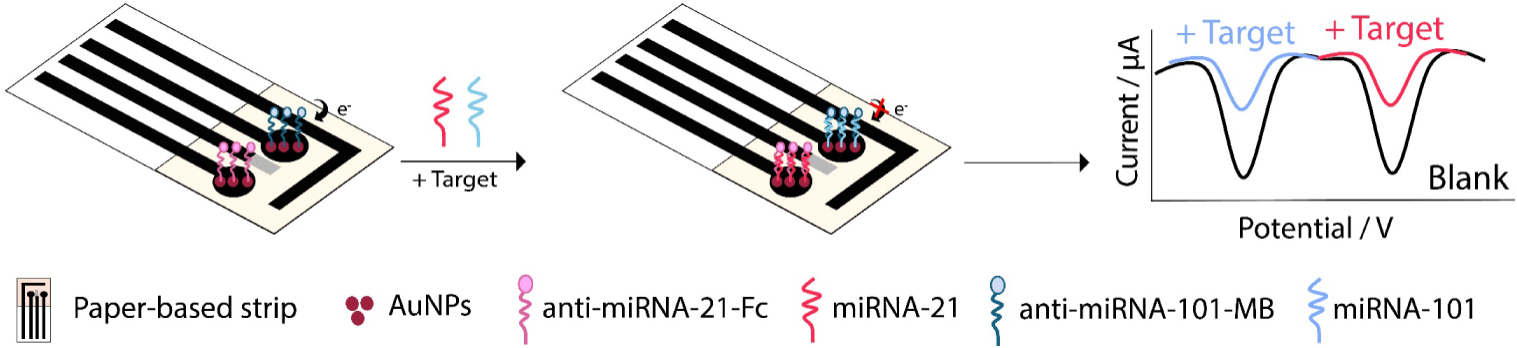
Schematic representation for the dual channel platform for the detection of miRNA-21 and miRNA-101 in TNBC.

Thus, the electrochemical signal decreases proportionally to the presence of miRNA. For all measurements, the percentage signal change was calculated according to eq 1, where I_0_ is the current in the absence of miRNA and I_target_ is the current in the presence of miRNA. The same procedure was used for both standard solutions and human serum samples.

1 $$\mathrm{Signal change}\;\left(\%\right)=\frac{I_{0}-I_{\mathrm{target}}}{I_{0}}\times 100$$

## Results and Discussion

### Electrochemical Characterization of the SPEs

The electrochemical performance and uniformity of the two bare WEs were first validated using cyclic voltammetry (CV). To obtain a comprehensive assessment, we employed two benchmark redox probes with distinct electron-transfer mechanisms. The outer-sphere couple (Fe­(CN)_6_^3–^/Fe­(CN)_6_^4–^) allowed us to assess surface-sensitive electron-transfer behavior at the SPEs, while the inner-sphere probe hexaammineruthenium­(III) chloride [Ru­(NH_3_)_6_]^3+^ was used as it is more representative of diffusion-controlled kinetics. Each probe was tested at 5 mM in 0.1 M KCl. Both WEs interrogated under identical experimental conditions across the full scan-rate range (0.02–0.5 V/s). An overview of the voltammetric responses obtained with these two systems is shown in Figure 2.

**Figure 2:**
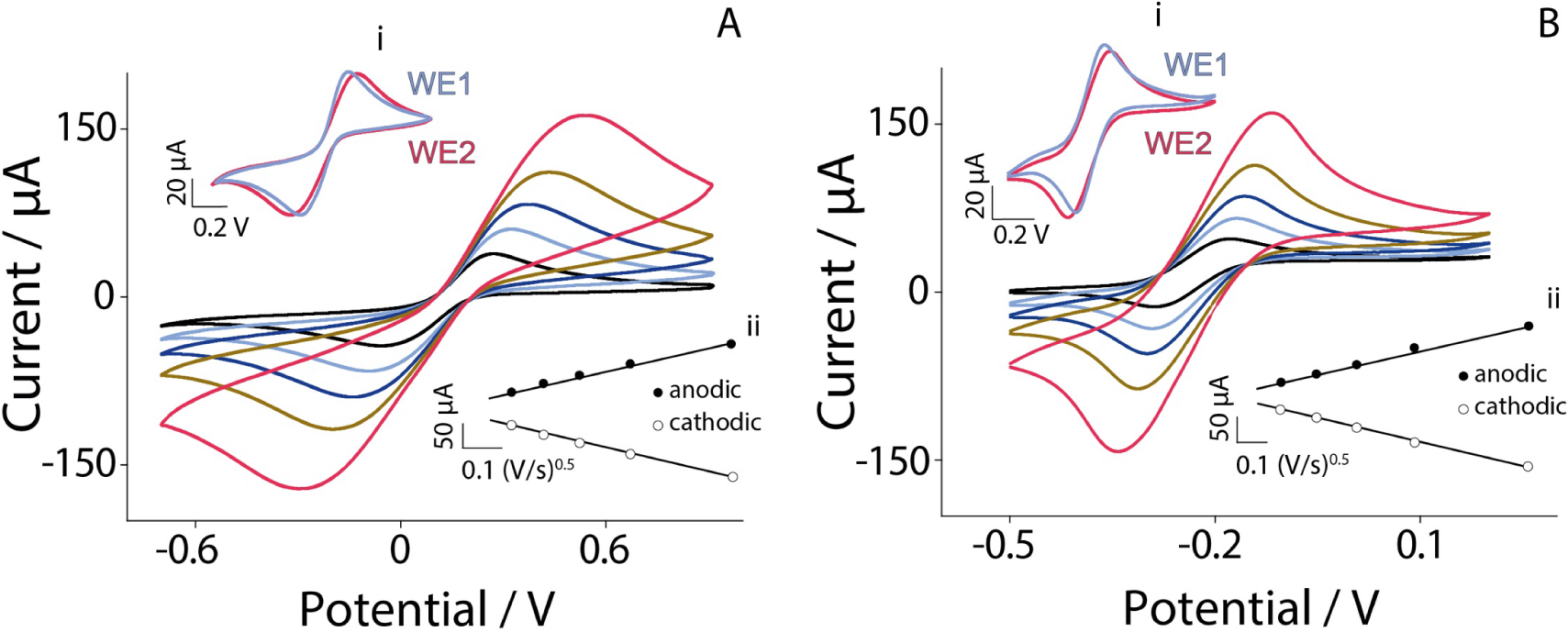
Electrochemical characterization of the dual-channel bare SPEs using (A) Fe­(CN)_6_^3–^/Fe­(CN)_6_^4–^ and (B) Ru­(NH_3_)_6_^3+^ in 0.1 M KCl over scan rates of 0.02–0.5 V·s^–1^. Insets (i) show representative voltammograms for WE1 and WE2 at an intermediate scan rate, while insets (ii) report the linear i_p_-(V/s)^0.5^ trends for anodic and cathodic peaks, demonstrating diffusion-controlled kinetics and reproducible electrode behavior.

As shown in Figure 2, the anodic (i_pa_) and cathodic (i_pc_) peak currents for [Fe­(CN)_6_]^3–^/^4–^ and [Ru­(NH_3_)_6_]^3+^ showed a linear dependence on the square root of the scan rate, confirming a diffusion-controlled process. This trend was confirmed by the linear regressions obtained for all oxidation and reduction peaks. The ferricyanide probe yielded i_pa_ = 180.08 + 10.78 v^1/2^ (R^2^ = 0.99) and i_pc_ = −179.31 – 10.41 v^1/2^ (R^2^ = 0.99). Ru­(NH_3_)_6_^3+^ showed similar behavior, with i_pa_ = 137.75 – 0.42 v^1/2^ (R^2^ = 0.99) and i_pc_ = −139.21 + 0.97 v^1/2^ (R^2^ = 0.99). The linear responses observed for both redox couples confirm that the system operates under diffusion-controlled kinetics for both channels, a key assumption for quantitative electrochemical sensing. This high degree of intradevice reproducibility is visually confirmed by the overlapping voltammograms for WE1 and WE2 at an intermediate scan rate (Figure 2, inset (i), which show matching peak positions and currents, thereby confirming electrode uniformity prior to surface modification.

### Analytical Characterization in Standard Solution and Serum

Following the electrochemical characterization, we evaluated the analytical performance of the dual-sensing platform. Increasing concentrations of miRNA-101 and miRNA-21 (0.1–1000 nM) were analyzed independently under the square-wave voltammetry (SWV) conditions described above, and the corresponding calibration curves were obtained both in PBS (pH 7.4) and in serum (Figure 3).

**Figure 3:**
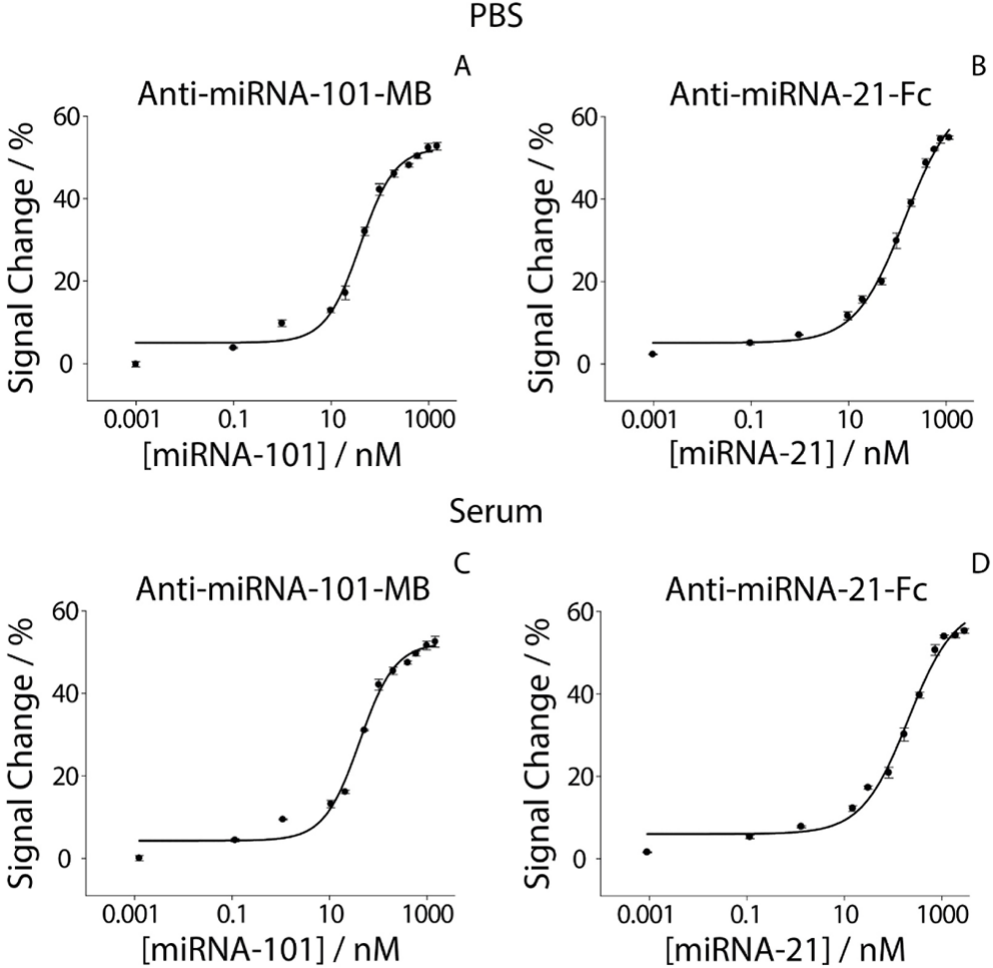
Calibration curves for miRNA-101 and miRNA-21 obtained by testing increasing target concentrations (0.1–1000 nM) in PBS (A, B) and in serum (C, D). Measurements were performed in triplicate (*n* = 3). SWV parameters: t_eq = 5 s, E_start = 0.7 V, E_end = −0.7 V, E_step = 0.001 V, amplitude = 0.01 V, frequency = 50 Hz.

Representative SWV traces obtained at increasing target concentrations are reported in Figure S1 in the (Supporting Information SI). In both channels, a progressive decrease of the corresponding redox peak current was observed as the target concentration increased, while the characteristic oxidation potentials of methylene blue (∼−0.3 V) and ferrocene (∼+0.4 V) remained well resolved, confirming the absence of electrochemical cross-talk between the two redox mediators. As shown in Figures 3A and 3B, both WEs exhibited a semilogarithmic sigmoidal correlation between the signal change (%) and the logarithm of the target concentration in PBS. In each case, a progressive increase in the signal change was observed over the tested concentration range, in line with the expected behavior of hybridization-based electrochemical assays. A comparable sigmoidal trend was also obtained in human serum (Figure 3C,D), confirming that the dual-channel configuration maintains reliable analytical performance even in an untreated complex matrix. The data were fitted using a Hill equation to account for the sigmoidal dose–response behavior, and the resulting coefficients of determination (R^2^), obtained from the nonlinear regression analysis, were above 0.99 for both targets in buffer and serum. The estimated limit of detection (LOD), operationally defined as the concentration corresponding to a 10% of the experimentally observed maximum signal change response. This threshold was selected because it was significantly higher than the baseline signal variability, exceeding the mean blank signal change (%) response (*n* = 5) plus three standard deviations (mean_blank SC% + 3SD), which were 3.2% for WE1 (MB-probe) and 2.6% for WE2 (Fc-probe), thereby ensuring statistically reliable discrimination from the background signal. Under these conditions, the operational LOD was 0.4 nM (miRNA-101) and 0.5 nM (miRNA-21) in PBS, while in serum was 0.6 nM and 0.4 nM, respectively. The dual biosensor’s repeatability, assessed at 50 nM for both targets using five independently fabricated devices (*n* = 5), yielded RSD values around 4%, demonstrating the robustness and precision of the device. These findings confirm the reliability of the dual-channel configuration and reinforce its relevance as a practical tool for multiplex detection of clinically important miRNA targets.

A comparison of reported nucleic acid detection platforms (Table 1) highlights the distinct advantages of the dual-channel paper-based sensor developed in this study. Most previously reported platforms focus on single-miRNA detection and often rely on complex amplification strategies, preconcentration steps, advanced nanomaterial conjugates, or enzymatic assays, sometimes requiring elevated temperatures, which can limit their practicality for POC applications. Detection limits in these systems range from nanomolar to attomolar levels.^39–41^ Multiplexed detection has been reported in a few cases, mainly for miRNA-21 and miRNA-155, using bulk carbon or graphene-based electrodes with sophisticated amplification strategies.^42–45^ In contrast, our proposed dual-channel sensor enables simultaneous detection of oncogenic miRNA-21 and tumor-suppressive miRNA-101, both in buffer and spiked human serum, without additional target or signal amplification or any further reagent addition steps. The platform achieves clinically relevant low-nanomolar LOD, while maintaining a short assay time (∼30 min at RT) and simple operational steps. This combination of multiplex capability, reproducibility, storage stability, clinically relevant sensitivity, POC-friendly design, and minimal sample preparation positions our system as a practical and scalable tool for TNBC-associated miRNA profiling, providing clear advantages over many existing nucleic acid detection platforms. Future improvements may incorporate functional nanomaterials, such as carbon nanofibers, metal–organic frameworks, or graphene-based nanostructures, to further enhance signal transduction and reduce the detection limit. Unlike amplification-driven or multilayer nanomaterial assembly strategies that significantly increase fabrication complexity, such integrations could be adapted to our paper-based architecture without substantially extending the probe immobilization or target detection protocol as previously reported.^46,47^ This approach would enable improved sensitivity while preserving the simplicity, speed, and POC compatibility of the current platform.

**Table 1:** Comparison of Reported Nucleic Acid Electrochemical Detection Platforms^a^

Platform	Type of disease	Type of target	Multiplexing	LOD	Assay time/temperature	Sample	Ref
MoS_2_/AuNPs/AgNW paper-based electrode with PtCuMOFs/DNA signal amplification	Cancer	miRNA-141, miRNA-21	YES	0.1 fM (PBS)	150 min/37°	PBS; spiked human serum	^27^
AuNPs-functionalized paper-based SPE; MB-tagged DNA probe	TNBC	miRNA-21	NO	1.2 nM (serum);	30 min/RT	PBS; spiked human serum	^37^
DSN-assisted signal amplification; MB-labeled DNA probe SPE	Cancer	miRNA-200a	NO	26 fM (PBS)	30 min/RT	PBS; spiked human serum	^39^
Paper-based Ag@Au core–shell nanoparticle on GQD nanoink PNA electrochemical sensor	Cancer	miRNA-21	NO	LOQ: 5 pM (human plasma)	5 min/37 °C	Spiked human plasma	^40^
Inkjet-printed Au/AuNPs paper electrode with thiolated ssDNA-21 SAM	Prostate cancer	miRNA-21	NO	0.35 fM (serum)	15 min/RT	Diluted fetal bovine serum	^41^
Carbon-fiber paper electrode functionalized with DNA probes; MIL-88(Fe)-NH_2_ MOF “signal carriers”	Breast cancer	miRNA-21, miRNA-155	YES	0.64 fM (miRNA-21)/0.54 fM (miRNA-155) (PBS)	70 min/37°	PBS; spiked human serum; cell lysate	^42^
DSN-coupled GNAs/AuNPs CP electrode	Lung and breast cancer	miRNA-21, miRNA-155	YES	21.4 aM (miRNA-21)/30.3 aM (miRNA-155) (PBS)	16 min/RT	PBS; human plasma samples	^43^
AuNP/RGO, AuNPs/MoS_2_ on carbon WE	Lung cancer	miRNA-21, miRNA-155	YES	12 nM (miRNA-21)/25.7 nM (miRNA-155) (PBS)	35 min/RT	PBS	^44^
AuNPs-modified LIG electrode with probes and SA-HRP immunoamplification	Nucleic acid methylation	m^6^ A-RNA, 5mC-ssDNA	YES	2.81 pM (m^6^ A-RNA)/9.53 pM (5mC-ssDNA)	45 min/37°	PBS, HeLa-derived spiked extracts	^45^
AuNPs-functionalized paper-based SPE with MB-DNA probe	Cancer	miRNA-2115–3p	NO	1 nM (PBS)	30 min/RT	PBS; spiked human serum	^48^
MB-ssDNA paper electrode	Lung cancer	miRNA-224	NO	0.6 nM (PBS)	30 min/RT	PBS; spiked human serum	^49^
Paper-based electrode modified with bulk MoS_2_ crystals or nanosheets	Lung cancer	miRNA-21, miRNA-155	NO	1.3 nM (miRNA-21)/2.3 nM (miRNA-155)	30 min/RT	PBS; diluted fetal bovine serum	^28^
AuNPs-functionalized paper-based screen-printed dual-WE with thiolated Fc- and MB-labeled DNA probes	TNBC	miRNA-21, miRNA-101 (dual detection)	YES	0.4 nM (miRNA-21)/0.6 nM (miRNA-101) (serum)	30 min/RT	PBS; spiked human serum	This work

a Abbreviations: Ag@Au, silver–gold core–shell nanoparticles; AgNW, silver nanowires; AuNPs, gold nanoparticles; CP, carbon paper; DSN, duplex-specific nuclease; Fc, ferrocene; GNAs, graphene nanoarrays; GQD, graphene quantum dots; LIG, laser-induced graphene; LOQ, limit of quantification; MB, methylene blue; MIL-88­(Fe)-NH_2_MOF, amino-functionalized iron-based metal–organic framework; MoS_2_, molybdenum disulfide; PBS, phosphate-buffered saline; PNA, peptide nucleic acid; PtCuMOFs, platinum–copper metal–organic frameworks; RGO, reduced graphene oxide; RT, room temperature; SA-HRP, streptavidin–horseradish peroxidase; SAM, self-assembled monolayer; SPE, screen-printed electrodes; ssDNA, single-stranded DNA; TNBC, triple-negative breast cancer; WE, working electrode.

### Simultaneous Detection of miRNA-101 and miRNA-21

Following the validation of single-analyte performance, we interrogated the multiplexing capability of our platform using a sample containing both miRNA-101 and miRNA-21 targets. The device enabled the simultaneous and independent quantification of both targets. To rigorously evaluate selectivity and potential cross-talk, we analyzed three distinct scenarios in both PBS and untreated human serum: (i) a mixture of 50 nM miRNA-101 and 200 nM miRNA-21, chosen based on the single-target assays (as described in Analytical Characterization in Standard Solution and Serum section) because these concentrations produced comparable levels of signal suppression in their respective channels, enabling a balanced evaluation of multiplex performance (Figure 4A,D); (ii) a sample containing 0.1 nM miRNA-101 and 800 nM miRNA-21 (Figure 4B,E); and (iii) a sample containing 800 nM miRNA-101 and 0.1 nM miRNA-21 (Figure 4C,F). Cases (ii) and (iii) were chosen to generate highly asymmetric conditions, where one miRNA produces a strong signal-off response while the other remains near the baseline, allowing us to challenge the platform under extreme dual-target scenarios.

**Figure 4:**
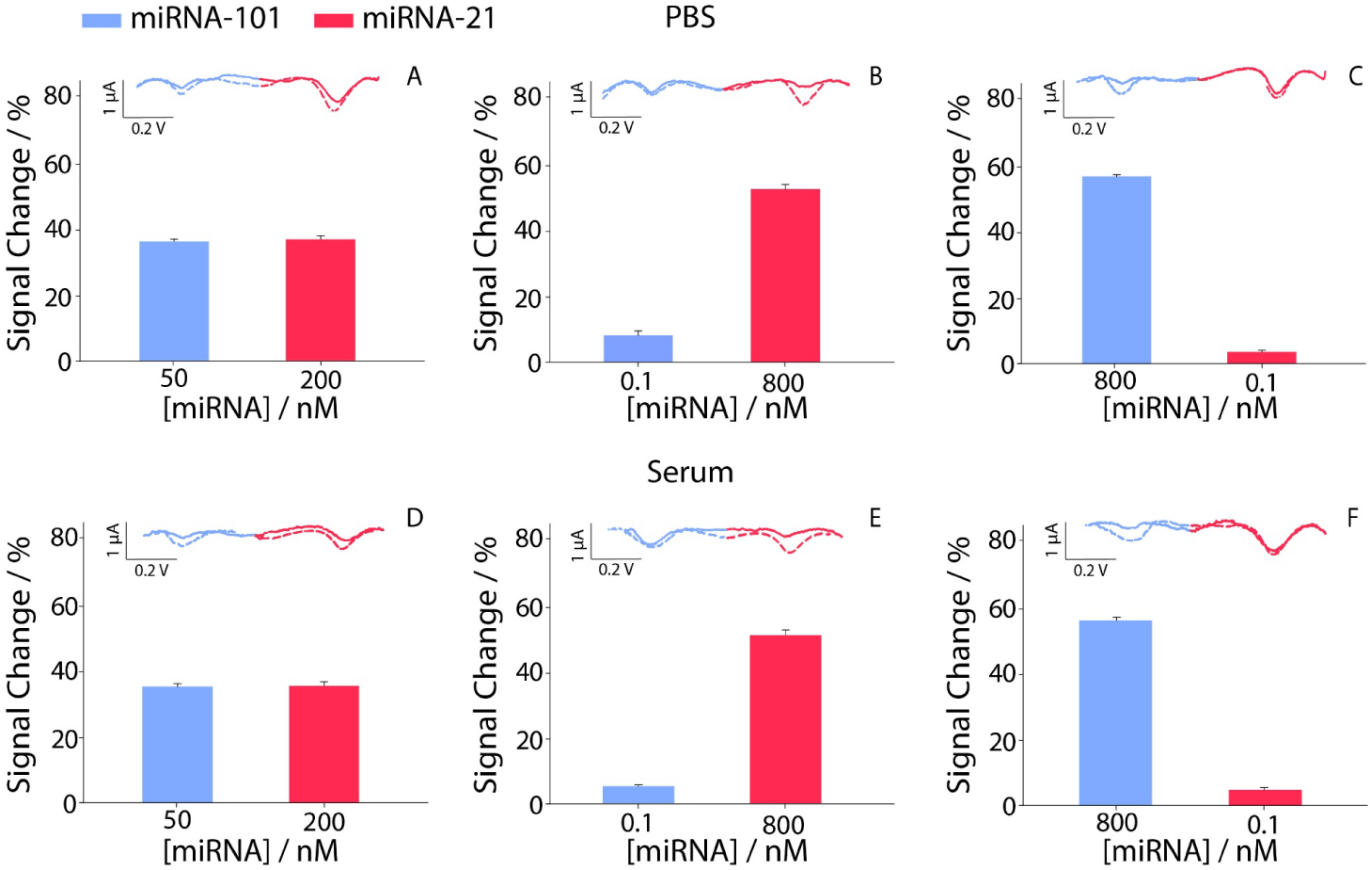
Simultaneous detection of miRNA-101 and miRNA-21 in PBS (A–C) and serum (D–F). Three experimental cases were evaluated: (A, D) mixed moderate concentrations; (B, E) low miRNA-101 (0.1 nM) and high miRNA-21 (800 nM) concentrations; (C, F) high miRNA-101 (800 nM) and low miRNA-21 (0.1 nM). Insets display representative SWV traces showing distinct signals from methylene blue (MB, miRNA-101 probe) and ferrocene (Fc, miRNA-21 probe) (*n* = 3).

In the mixed-miRNA samples, both WEs produced signal-off responses consistent with the presence of their complementary targets, confirming that the two redox reporters (methylene blue and ferrocene) can be monitored concurrently without interfering with each other. The concurrent signal-off responses at both WEs in the mixed-target samples confirmed successful, orthogonal monitoring through the methylene blue and ferrocene redox mediators without mutual interference. The platform’s molecular specificity was further confirmed under highly asymmetric target conditions, where one miRNA was present at a high concentration (800 nM) and the other at a much lower level (0.1 nM), representing a difference of 4 orders of magnitude. In these scenarios, only the WE carrying the complementary probe produced a significant signal-off response, while the nonspecific WE remained close to its baseline, i.e., the blank sample response. This selective behavior was preserved even when the concentration profile of the two targets was inverted, demonstrating that the dual-WE system maintains probe specificity and prevents cross-talk even in extreme dual-target mixtures. Importantly, this high-fidelity performance, characterized by strong multiplexing capability and minimal intertarget interference, was consistently maintained even in complex biological matrices, i.e., untreated human serum. This confirms the platform’s reliability in complex biological matrices. Overall, the device enables the simultaneous, specific, and cross-talk-free electrochemical detection of miRNA-101 and miRNA-21 from a single measurement.

### Specificity Studies

To assess the specificity of the developed platform, its selectivity was evaluated in the presence of potentially interfering, noncomplementary miRNAs associated with TNBC, namely miRNA-125 (5′-UCC CUG AGA CUU GUG A-3′),^50^ miRNA-200 (5′-UAA CAC UGU CUG GUA ACG AUG U-3′),^51^ and miRNA-205 (5′-GAU UUC AGU GGA GUG AAG UUC-3′).^52^ This evaluation was carried out in untreated human serum, using a constant concentration of 50 nM for each tested species. As shown in Figure S2A (WE1, MB-probe) and Figure S2B (WE2, Fc-probe) in SI, the signal changes recorded in the presence of the noncomplementary sequences were significantly lower than those obtained for the fully complementary targets. In both cases, the responses to the interfering miRNAs remained close to the baseline level, with signal deviations below 5%, indicating negligible nonspecific hybridization effects. These results confirm the high selectivity of the developed platform and demonstrate its ability to maintain specific target recognition even in complex biological matrices.

### Storage Stability and Batch-to-Batch Reproducibility

The storage stability of the dual-channel paper-based sensor was systematically evaluated under different storage conditions to assess their robustness for practical applications. After complete surface functionalization, the electrochemical response of each device was immediately recorded in the presence of 50 nM of each target miRNA. This response was defined as the initial signal response (Day 0) and used as the reference value for subsequent stability assessment. The sensors were then stored under four different conditions designed to independently evaluate the effects of temperature and humidity: (i) at 4 °C in Petri dishes in a dry state, (ii) at 4 °C in Petri dishes with a buffer drop applied, (iii) at RT in Petri dishes in a dry state, and (iv) at RT in vacuum-sealed bags in a dry state. At each time interval (days 1, 2, 3, and 7), the same analytical measurement was repeated under identical experimental conditions, and the corresponding signal change (%) was calculated for both channels. Signal retention (%) was then determined by normalizing the signal change at each time point to the initial Day 0 value (Table S1 in SI). Under dry storage at 4 °C, both channels showed excellent stability over 1 week, with signal retention of 94.4 ± 1.7% (MB) and 93.3 ± 1.9% (Fc) at day 7, indicating a signal decrease below 7%. This behavior is consistent with the improved preservation of thiolated DNA monolayers on AuNP-modified surfaces at lower temperature, where molecular mobility and oxidation-driven degradation processes are reduced.^53,54^ In contrast to the excellent stability observed under dry storage, wet storage at 4 °C resulted in a markedly accelerated signal decay. Within 48 h, the analytical response decreased by more than 20%, and beyond day 2 no reproducible electrochemical response could be obtained precluding further quantitative evaluation. The presence of an aqueous environment likely promotes increased interfacial molecular mobility and facilitates partial desorption or reorganization of the thiolated DNA/MCH layer, thereby compromising the integrity of the sensing interface.^54,55^ When the storage temperature increased to RT, no significant signal deterioration was observed within the tested period. The high stability can be attributed to the absence of enzymatic or target amplification steps, which are often sensitive to storage conditions and temperature variations. Limiting exposure to oxygen and humidity, as well as to airborne contaminants, further mitigates interfacial degradation processes and improves long-term stability. Accordingly, vacuum-sealed dry storage at room temperature enhanced signal retention, yielding 90.8 ± 1.3% (MB) and 90.1 ± 1.4% (Fc) on day 7. These findings indicate that simple vacuum packaging represents an effective strategy to preserve the functional integrity of the sensing interface during RT storage. These results support the robustness and reliability of the proposed platform and highlight its potential suitability for POC applications in multiplexed miRNA detection.

To further assess fabrication reliability, batch-to-batch reproducibility was evaluated using independently printed electrode batches prepared on different days. These batches were subsequently tested either on the day of fabrication and on different testing days under identical experimental conditions at 50 nM target concentration, to assess both production and operational variability. The electrochemical responses demonstrated consistent signal intensity and target discrimination across all conditions (Figure S3 in SI). The calculated relative RSD% values were 2.3% for miRNA-101 and 2.4% for miRNA-21, indicating minimal interbatch variation. These findings confirm the robustness and reproducibility of the in-house screen-printing and functionalization processes, supporting the scalability and translational potential of the proposed paper-based multiplex platform.

## Conclusions

This technical note demonstrates a clinically meaningful dual-channel electrochemical paper-based biosensor capable of simultaneously detecting miRNA-21 and miRNA-101 in circulating biofluids. By integrating an oncogenic and a tumor-suppressive miRNA within a single platform, the approach offers significantly stronger diagnostic contrast and biological resolution than traditional single-biomarker formats. The incorporation of redox-tag multiplexing into a paper-based electrochemical architecture represents a notable advancement, overcoming the constraints of earlier single-channel paper sensors and enabling true multimarker interrogation at the point of care. Overall, this platform advances the field of accessible molecular diagnostics by providing a low-cost, rapid, and portable system that maintains analytical performance comparable to laboratory-based methods. Its multiplex design directly addresses challenges of tumor heterogeneity and enhances the clinical interpretability of circulating miRNA profiles. While the present multiplexing strategy relies on spatially separated working electrodes, expansion to additional biomarkers is technically feasible within the current screen-printing architecture. Future developments may incorporate mixed probe immobilization and additional redox reporters to further increase multiplexing density. These strategies will enable scalable, higher-order miRNA signature profiling while preserving the simplicity and POC compatibility of the platform. Beyond the immediate application to breast cancer, the work establishes a scalable and versatile foundation for future multiplexed paper-based biosensors capable of capturing broader miRNA signatures and supporting precision oncology, early detection, treatment monitoring, and global diagnostic equity.

## Supplementary Material



## Notes

Representative square-wave voltammograms recorded in untreated human serum at increasing concentrations of miRNA-101 and miRNA-21 (Figure S1), selectivity studies in untreated human serum (Figure S2), the storage stability of the dual paper-based sensors (Table S1), and the batch-to-batch reproducibility (Figure S3) (PDF)
