# Supplementary material for: Dual Paper-Based Electrochemical Device for Multiplex Detection of Triple-Negative Breast Cancer miRNA Signatures
Source: Anal Chem. 2026 May 22;98(22):15968–77. doi: 10.1021/acs.analchem.5c07951 (PMC13261615; doi:10.1021/acs.analchem.5c07951)
Supplement: Supplementary file 1 [file ac5c07951_si_001.pdf]

Supporting information

Dual paper-based electrochemical device for multiplex detection of triple-negative breast cancer miRNA signatures

*Alessandra Glovi[a,b], Panagiota M. Kalligosfyri[b], Antonella Miglione[c], Sima Singh[b], Wanda Cimmino[b], Stefania Cocco[d], Umberto Malapelle[c], Antonio Giordano[e,f], Michelino De Laurentiis[d], Stefano Cinti[b,e]**

[a]Clinical and Translational Oncology, Scuola Superiore Meridionale (SSM), 80138, Naples, Italy

[b]Department of Pharmacy, University of Naples Federico II, 80131, Naples, Italy

[c]Department of Public Health, University Federico II of Naples, 80131, Naples, Italy

[d]Department of Breast and Thoracic Oncology, Istituto Nazionale Tumori IRCCS "Fondazione G. Pascale", 80131, Naples, Italy

[e]Sbarro Institute for Cancer Research and Molecular Medicine, Center for Biotechnology, College of Science and Technology, Temple University, Philadelphia, Pennsylvania 19122, USA

[f]Department of Medical Biotechnologies, University of Siena, 53100, Siena, Italy

*Corresponding author: stefano.cinti@unina.it

Table of content

Figure S1. Representative square-wave voltammograms at increasing concentrations of miRNA-101 and miRNA-21 in untreated human serum Page S2

Figure S2. Selectivity study in untreated human serum Page S3

Table S1. Storage stability under different temperature and humidity conditions Page S3

Figures S3. Batch-to-batch reproducibility of the dual-channel platform Page S4

Representative square-wave voltammograms (SWVs) at increasing concentrations of miRNA-101 and miRNA-21 in untreated human serum

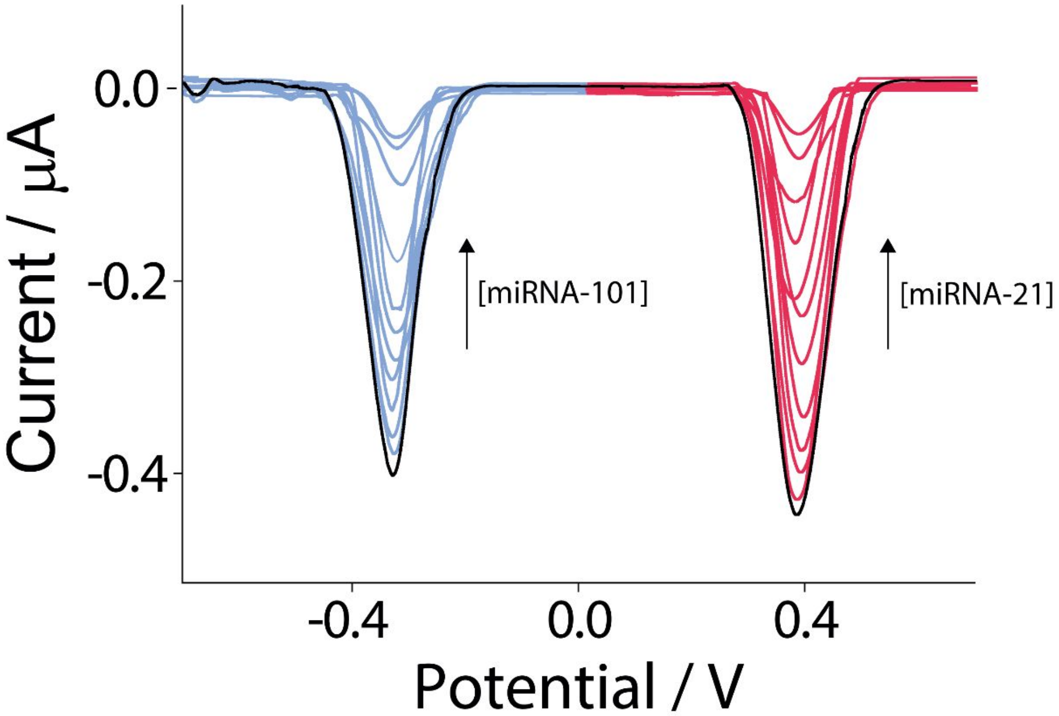

Figure S1. Representative SWVs obtained in untreated human serum before target addition (black curve) and after the addition of increasing concentrations (0.1–1000 nM) of miRNA-101 and miRNA-21. Each SWV curve was recorded in a single measurement of the same: the blue color is used to highlight the MB peak (~−0.3 V), while the red color is used to highlight the Fc peak (~0.4 V) corresponding to increasing miRNA-101 and miRNA-21 concentrations respectively. The progressive decrease of both peaks upon target addition is consistent with the signal-off sensing mechanism.

Selectivity study

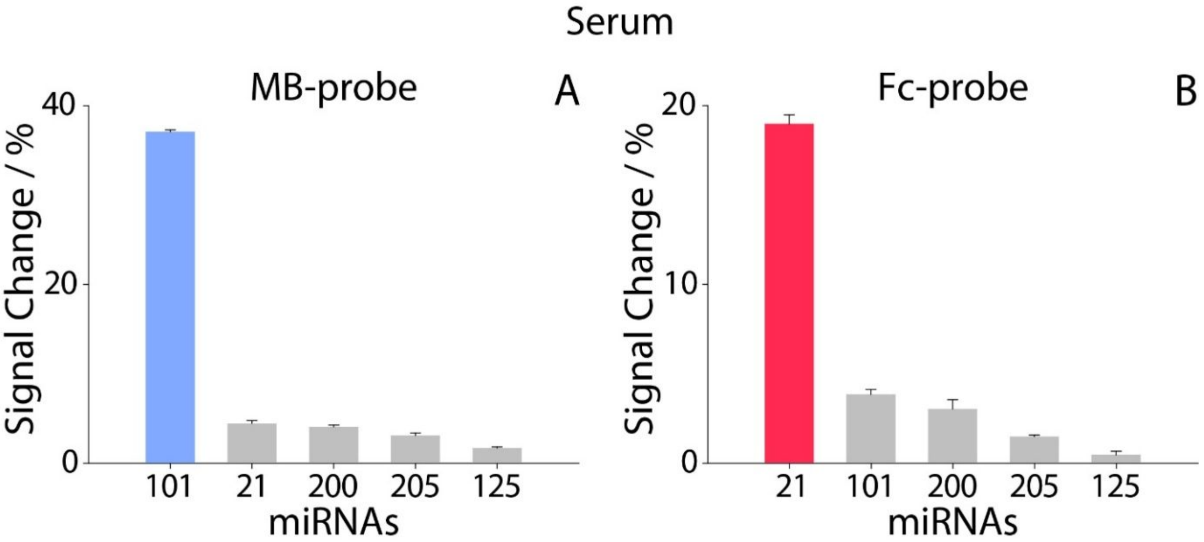

Figure S2. Selectivity of the dual-channel platform evaluated in untreated human serum. Signal change (%) recorded at (A) WE1 (MB-probe) and (B) at WE2 (Fc-probe) in the presence of 50 nM of the fully complementary targets (miRNA-101 for WE1 and miRNA-21 for WE2), the non-complementary target (miRNA-21 for WE1 and miRNA-101 for WE2), and additional interfering miRNAs (miRNA-125, miRNA-200, and miRNA-205).

Storage stability of the dual-channel platform

Storage condition	Storage day							
	Day 1		Day 2		Day 3		Day 7	
	WE1 (MB-probe)	WE2 (Fc- probe)	WE1 (MB-probe)	WE2 (Fc- probe)	WE1 (MB-probe)	WE2 (Fc- probe)	WE1 (MB-probe)	WE2 (Fc- probe)
	Signal Retention (%) = (Signal of storage condition /Signal Day 0) × 100							
4° / WET	83.4 % ± 0.7	77.6 % ± 0.5	78.8 % ± 0.8	71.9 % ± 1.1	N/O	N/O	N/O	N/O
4° / DRY	98.6 % ± 0.8	97.8 % ± 0.9	95.4 % ± 1.3	94.4 % ± 1.2	94.4 % ± 1.5	93.6 % ± 1.3	94.4 % ± 1.7	93.3 % ± 1.9
RT / DRY	93.5 % ± 0.9	97.0 % ± 1.3	93.9 % ± 1.0	91.9 % ± 1.1	86.8 % ± 1.3	83.5 % ± 1.5	84.0 % ± 1.5	79.0 % ± 2.5
RT / DRY / VACUUM	98.9 % ± 0.5	97.5 % ± 1.1	97.3 % ± 0.8	96.1 % ± 1.4	95.4 % ± 1.3	93.5 % ± 1.3	90.8 % ± 1.3	90.1 % ± 1.4

RT: Room temperature; N/O: Not obtained

Table S1. Storage stability of the dual-channel paper-based sensor under different temperature and humidity conditions: (i) at 4 °C in Petri dishes in a dry state, (ii) at 4 °C in Petri dishes with a buffer drop applied, (iii) at RT in Petri dishes in a dry state, and (iv) at RT in vacuum-sealed bags in a dry state. Measurements were performed at days 1, 2, 3, and 7 after storage in the presence of 50 nM target miRNAs. Values are reported as mean of ± standard deviation (n = 5) for WE1 (MB-probe) and WE2 (Fc-probe).

Batch-to-batch reproducibility of the dual-channel platform

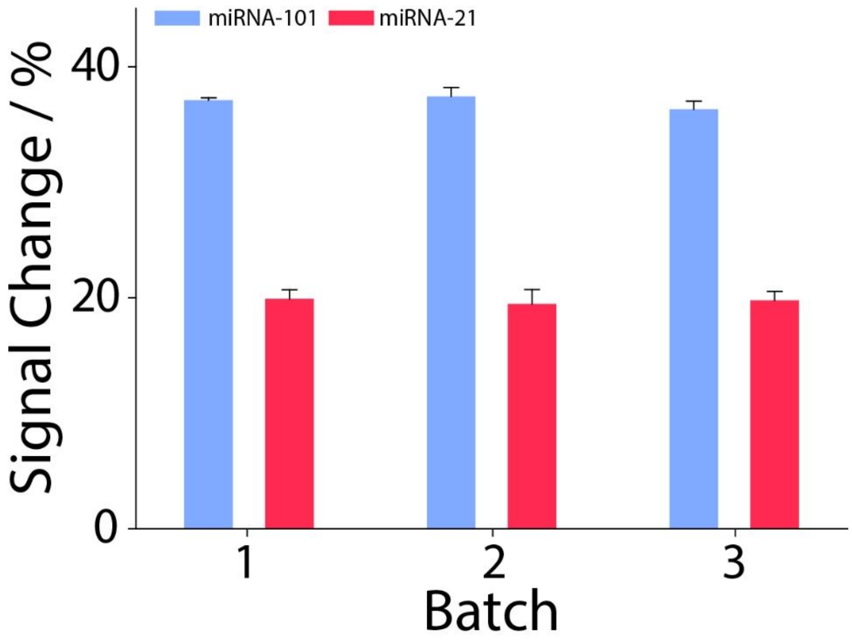

Figure S3. Batch-to-batch reproducibility of the dual paper-based electrochemical platform. Signal change (%) recorded for miRNA-101 and miRNA-21 at 50 nM using independently fabricated electrode batches (Batch 1–3). Measurements were performed under identical experimental conditions (n = 5). The consistent signal intensity across batches confirms minimal inter-batch variability.
